# Modelling Human Physiology on-Chip: Historical Perspectives and Future Directions

**DOI:** 10.3390/mi12101250

**Published:** 2021-10-15

**Authors:** Sirjana Pun, Li Cai Haney, Riccardo Barrile

**Affiliations:** 1 Department of Biomedical Engineering, College of Engineering and Applied Science, University of Cincinnati, Cincinnati, OH 45221, USA; punsa@mail.uc.edu (S.P.); haneyld@mail.uc.edu (L.C.H.); 2 Center for Stem Cell and Organoid Medicine, Cincinnati Children’s Hospital Medical Center, Cincinnati, OH 45221, USA

**Keywords:** Organ-on-Chip, microphysiological systems, personalized medicine, technological trajectories, precision medicine, iPSc, microfluidic, bioprinting, Lung-on-Chip, Brain-on-Chip

## Abstract

For centuries, animal experiments have contributed much to our understanding of mechanisms of human disease, but their value in predicting the effectiveness of drug treatments in the clinic has remained controversial. Animal models, including genetically modified ones and experimentally induced pathologies, often do not accurately reflect disease in humans, and therefore do not predict with sufficient certainty what will happen in humans. Organ-on-chip (OOC) technology and bioengineered tissues have emerged as promising alternatives to traditional animal testing for a wide range of applications in biological defence, drug discovery and development, and precision medicine, offering a potential alternative. Recent technological breakthroughs in stem cell and organoid biology, OOC technology, and 3D bioprinting have all contributed to a tremendous progress in our ability to design, assemble and manufacture living organ biomimetic systems that more accurately reflect the structural and functional characteristics of human tissue in vitro, and enable improved predictions of human responses to drugs and environmental stimuli. Here, we provide a historical perspective on the evolution of the field of bioengineering, focusing on the most salient milestones that enabled control of internal and external cell microenvironment. We introduce the concepts of OOCs and Microphysiological systems (MPSs), review various chip designs and microfabrication methods used to construct OOCs, focusing on blood-brain barrier as an example, and discuss existing challenges and limitations. Finally, we provide an overview on emerging strategies for 3D bioprinting of MPSs and comment on the potential role of these devices in precision medicine.

## 1. Introduction

The modern drug development process has adopted a multitude of promising in vitro approaches to accelerate and improve the success rate of drug discovery [1]. Advances in computational biology made over the last century have led to the establishment of in silico techniques which are capable of conducting virtual drug screenings and that have shown their potential to design and identify new compounds and accurately predict clinical risk. Artificial Intelligence (AI) and related technologies are increasingly prevalent in pharmaceutical industries to help streamline timelines associated with bringing a new drug to market. Nevertheless, empirical testing of drugs still represents a vital step for the validation of new mechanistic hypothesis and computer-based predictions of drug efficacy and toxicity.

The discrepancy between results obtained in preclinical studies, which are typically derived from animal experimentation, and clinical outcomes is the root cause of drug failure across diverse disease areas [2]. The deteriorating attrition rate, which currently sees less than one in ten new drugs obtain approval [3,4], highlights the fact that animal models are simply not good predictors of the human biology. Therefore, there is an urgent need to develop more predictive and reliable [5] human-relevant models that are able to faithfully recapitulate the physiological microenvironment of human tissues and organs to provide better alternatives to animal testing.

Recent advancements in bioengineering have led to the development of complex cell culture models, frequently referred to as Microphysiological systems (MPSs) or Organs-on-Chips (OOCs) [6]; these microfluidic cell culture devices are composed of optically clear plastic, glass or flexible polymers and contain perfused hollow microchannels populated by living cells. Such microfluidic devices can be used to create “tissue chips” with channels lined by multiple cell types to combine, for example, tissue-specific parenchymal and vascular microchannels, thereby recreating tissue–tissue interfaces that are crucial for reconstituting organ-level structures and functions [7].

This review aims to trace potential future designs and applications for MPSs and OOCs. To predict the evolution of these technologies, however, we believe it is essential to retrace their past evolution. MPSs and OOCs represent novel bioengineering approaches to model the human tissue microenvironment (TME) at an unprecedented level of control [8]. However, the basic concept of reconstituting cell and tissue function in vitro by culturing human cells in a controlled microenvironment is not new, as it has been dominating the field of biomedical research for over a century. An adequate historical perspective is necessary to understand where the field comes from and where it is going. Therefore, in this review, we will first provide a brief overview of commonly used 2D and 3D cell culture methods and discuss some of the critical historical milestones that marked the transition of mechanobiology from a descriptive field to an experimental science paving the way toward the modern concept of OOCs, as well as the relevance of synergistic partnerships between industry and regulatory bodies in building of predictive OOCs and MPSs. In an effort to predict potential future designs, we will review some of the most recent articles describing novel OOCs models of the lungs and brain, with the goal of identifying emerging elements of design, and predict future applications of these vascularized models. Finally, we will review the most recent 3D bioprinting approaches to biofabricating MPSs, that will open the door to on-demand and personalized models, which may replace many preclinical steps in future drug trials.

### Terminology

The terms MPSs and OOCs are frequently used as synonyms, both referring to microfluidic-based in vitro models designed to capture the dynamic biochemical microenvironment of living organs. OOCs are “called ‘chips’ because they were originally fabricated using methods adapted from those used for manufacturing of computer microchips” such as soft-lithography [7]. Therefore, this review will use the term “OOC” when referring to a device that was fabricated using traditional microfabrication methods, including soft-lithography and photo-lithography [9]. The term “MPS” will be used when referring to systems developed using alternative biofabrication strategies such as bioprinting.

## 2. Modelling Human Tissue Microenvironment in 2D and 3D Cell Cultures

A steadily increasing awareness across pharmaceutical companies and regulatory bodies (such as the FDA) of the limitations intrinsic to existing preclinical models has inspired a tremendous amount of effort aimed towards the development of alternative methods for studying the safety and biological effects of drugs, chemicals, agents, and other substances. The high clinical failure rate in drug development compounded with the high costs of animal research and some key ethical issues including animal welfare concerns have [5] fuelled the need to refine, reduce, and eventually replace current animal testing [10]. In vitro models of human cell and tissue-functions represent a formidable alternative to animal models for both studying the fundamental molecular principles that regulate our body and assessing of drug efficacy, as well as chemical safety in an efficient and economical manner.

### 2.1. Conventional 2D Models

**Figure 1:**
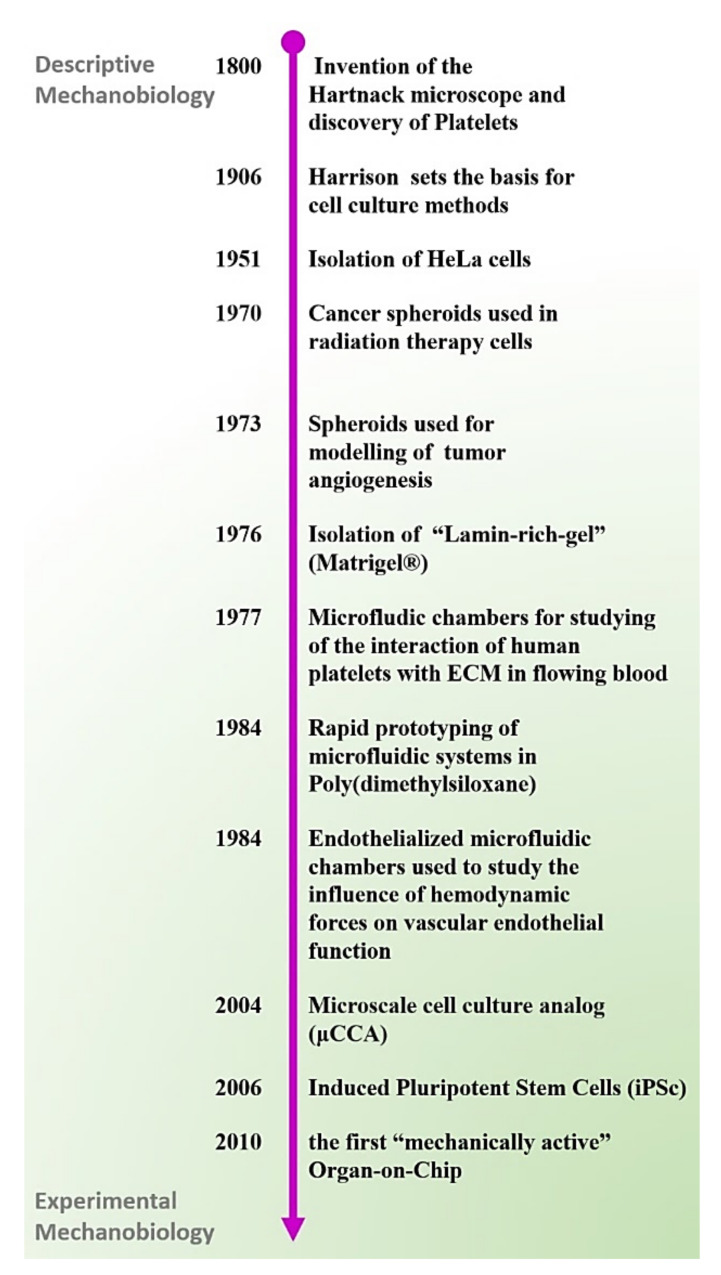
Historical milestones that signed the transition of mechanobiology from a descriptive field to an experimental science and paved the way toward the modern concept of Organ-on-Chip.

The most common cell culture methods draw from studies on the action of serum on fibroblast cells and the development of novel synthetic cell culture media [11,12] (Figure 1). Since the isolation and expansion of cancer cells from a cervical tumour (HeLa), culturing cells in a two-dimensional (2D) format has remained the predominant methodology for in vitro cell growth and expansion [13]. In adherent 2D cultures, cells grow in a flat configuration and eventually form a tight monolayer where individual cells are lined with junctional structures such as adherents and tight junctions that are central to barrier-function of epithelial and endothelial tissues. Two-dimensional cell cultures are relatively simple to perform, affordable and, when combined with robotic instrumentation, effective for screening large molecular libraries of drugs of unknown biological activity [14]. Unfortunately, 2D cell cultures do not effectively recapitulate the spatial organization that occurs in vivo which is central to promoting physiological cell–cell interaction and communication. Such interactions are ultimately responsible for cell differentiation, proliferation, vitality, expression of genes and proteins, responsiveness to stimuli, drug metabolism and other cellular functions [15].

Another drawback of 2D culture is that the cells in the monolayer have unlimited access to the ingredients of the medium such as oxygen, nutrients, metabolites and signal molecules. Multiple lines of evidence indicate that limited cell–cell contact and altered in vitro cell signalling networks can result in major discrepancies between the data acquired from two-dimensional in vitro versus in vivo research and therefore, data produced in 2D cell culture are often poorly translatable [16,17]. Finally, one drawback of traditional static cell culture for testing of drug candidates is that when cells are cultured in static conditions, the effective concentration of soluble molecules, including drugs and hormones, is constant and regulated only by the metabolic activity of the cell types present in the dish. Therefore, when cultured in static conditions, human cells are exposed to a constant concentration of drugs and growth factors as opposed to what occurs in the human body where multiple cell types operate synergistically within the same tissue to regulate endogenous tissue-function.

### 2.2. 3D Models: Spheroids and Organoids

Three-dimensional (3D) cell culture models have been central to the identification of environmental factors that drive cell migration, proliferation and differentiation during embryonic organ-morphogenesis, angiogenesis, or the progression of diseases such as cancer. Migration of stem cells and folding of the germinal layers during gastrulation as well as the angiogenic sprouting of blood vessels and migration of metastatic cells in adult tissues are “all cases of higher-order cell processes that are inherently 3D” that cannot be reproduced when cells are growing in a 2D configuration [18].

When cultured in optimized 3D matrixes (hydrogels), proliferating cancer cells and healthy pluripotent stem cells are able to self-organize in 3D clusters. Spheroids are cell clusters constituted by a single cell type and may not require scaffolding to an extracellular matrix. Given their easy-to-use protocols and microplate formats compatible with automation and screenings of multiple compounds, spheroids have been applied to several cell types and applied to experimental cancer research as well as oncology drug screening [19]. The utility of spheroids in drug development was first demonstrated in 1970 to recapitulate the functional phenotype of human cancer cells and their responses to radiotherapy [20,21]. 3D culture of cancer cell lines was also performed in order to study the angiogenic potential of multiple cancer cell lines, with methods initially developed by Folkman and colleagues over fifty years ago, and is currently broadly adopted to study the biology of vascularized tumours, including mechanisms regulating cancer dormancy and metastatic potential and their therapeutic implications [22,23,24].

The term “organoids” has been in use for over 50 years to refer to a multitude of methods developed for culturing animal cells in a 3D environment [25]. While the choice of biomaterials for 3D cell cultures had been initially limited to agar and collagen I, the isolation of a “laminin-rich gel” in the late 1970s and, the more recent (2005) commercialization of Matrigel^®^, has allowed researchers to further expand the use of 3D cell culture methods to a growing number of applications, including drug testing [26,27]. The term “organoids” is currently used for describing complex biological structures, harbouring a limited population of self-renewing stem cells that can differentiate into organ-specific cell types [28]. Organoid models are considered physiologically relevant systems and far superior to conventional 2D cell cultures in many ways. For example, when growing in 3D hydrogels, spheroids and organoids can develop gradients of oxygen, nutrients, metabolites, and soluble signals, thus creating (or differentiating into) heterogeneous cell populations that are not simple to obtain when cells are growing in a planar configuration. Healthy and diseased organoid models have been developed successfully from various organs, including the intestine, liver, lungs, and brain, demonstrating the tremendous potential of these 3D models for fundamental research and clinical applications including the study of organ development and human diseases [29].

One applicative example of organoid models used in drug development comes from a recent pilot-study performed using a colon cancer biobank in which the chemotherapeutic potential of over 80 compounds was tested [30]. Transcriptomic analysis confirmed the anticipated and direct association between gene-response to individual drugs, thereby supporting the possibility of using organoid biobanks for high-throughput screening of potential therapeutic molecules in colon cancer. Similar studies performed on organoid models of cystic fibrosis have further demonstrated the possibility of using patient-derived pulmonary organoids for the identification of optimal treatments [31]. While future studies will need to first demonstrate the accuracy of these predictions [32], current results suggest that soon these methods could be applied for predicting individual drug-response.

When compared to traditional 2D methods, 3D cell cultures better mimic the natural tissue architecture of human tissues. Organoids and spheroids, however, are typically cultured in static conditions and cannot capture the dynamic nature of animal TME where cells evolved countless mechanisms to react and adapt to mechanical cues generated by continuous blood flow, muscle contraction, change in substrate composition, and stiffness. Static cell culture methods, therefore, are not always adequate for recapitulating the dynamic nature of human TME. Multiple research papers have clearly demonstrated the impact of an altered blood flow dynamic on vascular homeostasis [33,34], including immune cell extravasation, blood clotting, as well as metastatic cell extravasation [35], all of which are biological processes of relevance for drug testing purposes that cannot be effectively modelled in static conditions [36,37].

## 3. Dynamic Control of the Cell Microenvironment

The emerging concept of microengineering organ-function in vitro (a steppingstone for OOCs) via culturing of human cells in a controlled and dynamic microenvironment builds on important milestones that mark the history of mechanobiology. Here, we describe and comment on some of the most remarkable scientific achievements that, in our opinion, signed the transition of mechanobiology from a descriptive field to an experimental science and paved the way toward the modern concept of Organ-on-Chip.

### 3.1. Mechanobiology: From a Descriptive Field to Experimental Science

As part of the natural developmental program of plants and animals, cells mature to adapt and react to specific mechano-stimulatory triggers. Mechanobiology has emerged in the last 20 years as a new scientific discipline aiming to elucidate the roles and mechanisms of mechanical forces in living organisms, but the possibility that mechanical forces can influence (or drive) tissue morphogenesis was postulated over a century ago [38,39]. Interestingly, the progresses made in the field of mechanobiology appear to be closely connected to the development of enabling technologies.

One of the earliest and probably most emblematic mechanobiology studies concerns the relationship between blood flow and thrombus formation which dates back to the 19th century when Bizzozero, an Italian pathologist, demonstrated the existence and function of platelets, “the third circulatory component of the blood” [40,41]. Although other scientists had previously described the process of blood-clotting occurring in blood samples withdrawn from animals and analysed in static conditions, Bizzozzero had the intuition that the dynamic mechanisms regulating blood-clotting may be different in ex vivo and in vivo conditions. Through his pioneering work, Bizzozzero discovered and carefully described the function of platelets under flowing conditions and the relationship between platelet adhesion, aggregation, and subsequent fibrin formation of blood clots. It would not be possible to obtain similar results without the use of “advanced microscopes” available to Bizzozzero and other scientists and medics at the end of the 19th century. The “Hartnack” microscope, a cutting-edge scientific instrument in the 1800′s that Bizzozzero “engineered” for controlling heath (37 °C), humidity, and light exposure was an enabling tool for the Italian pathologist who successfully adopted, for the first time, a “live imaging” approach to the study of hemodynamics. Unfortunately, given the scarcity of in vitro models of the time, it was impossible for Bizzozzero to confirm similar results in a human relevant system.

Nearly 90 years later, the invention of “perfusion chambers” enabled researchers to perform similar experiments with human blood [42]. The combined use of live imaging techniques with engineered perfusion chambers allowed advanced investigation into the mechanisms regulating platelet rolling, adhesion, and aggregation on immobilized ligands, and provided critical insights into shear-dependent receptor-ligand interactions [43,44,45], as well as to evaluate thrombus formation and antithrombotic agents under blood flow conditions [46,47]. Other mechanobiology studies conducted at the beginning of the 20th century include the work of the surgeon and anatomist, J. Wolff, who observed that trabeculae matched the principal stress lines in bones caused by daily physical loading and during the healing of fractured bones [48]. A few years later, mechanical forces were also proposed as major determinants in the organogenesis during embryonic development [39]. These initial observations, mostly conducted using microscopy as the only investigative technique, provided the basic knowledge to formulate new theories that challenged contemporary concepts on the role of physical forces in regulating the form and function an animal body. However, for a long time, the lack of adequate tools remained the main roadblock to validate animal data and prove these new theories in human relevant systems.

### 3.2. Cells and Tissues on-Chip

Microfluidic tools developed during the 20th century enabled a growing number of researchers to obtain novel mechanistic insights into the role of mechanical forces in vascular biology. For instance, in 1970, glass models of bifurcated aneurysms were used to demonstrate that the blood flows under a turbulent regime near the vascular region of an aneurysm [49]. Further development of microfluidic technologies, combined with the use of biocompatible materials, permitted other groups to bioengineer microfluidic constructs harbouring human cells, later referred to as “Cells-on-Chip” [50]. These devices were instrumental to demonstrate the relevance of laminar flow in maintaining tissue-homeostasis and the role of turbulent blood flow in triggering the inflammatory response of endothelial cells [51,52,53]. Models of blood vessels containing living endothelial cells were also used to mimic the effect of pulsating blood flow on endothelial and smooth muscle, demonstrating an important role for shear stress in regulating cell proliferation, and expression of inflammatory markers [54]. While the ability of fabricating biocompatible constructs for modelling biomechanical forces was, for a long time, limited to a small group of specialized laboratories, the invention of an optically transparent and economic silicon material (PDMS) in the late 1990s, combined with the use of soft lithography and precise mechano-actuators [55,56], allowed a growing number of researchers to expand their study in the field of mechanobiology. It was demonstrated, for example, that mechanical stretching caused by the physiological pulsatile blood flow, modulates the smooth muscles proliferation and secretion of extracellular metalloproteases [57,58], an observation that could explain the progressive weakening and eventual rapture of the vascular wall near a bulge of an aneurysm. The abovementioned studies are only some of the examples of application of Cells-on-Chip devices that were used for obtaining invaluable insights into the physiology of vascularized tissues.

### 3.3. The First Organ-on-a-Chip

Since the end of the 20th century, several additional studies have further expanded on the possibility of adopting microfluidic solutions for harnessing the biomechanical forces that govern tissue and organ functions in a human body. The concept of adapting microfluidic technology for modelling organ and systemic-level function of human physiology or disease was first published in 2004 when Dr. Shuler and colleagues demonstrated that a “microscale cell culture analog (μCCA)”, consisting of three parallel microfluidic chambers micro-fabricated using lithography-etching techniques and seeded with lung and liver cells, could be potentially used for recapitulating the systemic interaction between the human lungs and liver. This device was also interfaced with an oxygen sensor that could provide real-time measurements of cell metabolic activity in response to specific treatments. Results of this study led the authors to conclude that “when used in conjunction with a physiologically based pharmacokinetic” this μCCA could represent a valuable “human surrogate for predicting human response in clinical trials” [59]; this study was a remarkable achievement in the quest for finding better alternatives to animal models used in preclinical studies.

**Figure 2:**
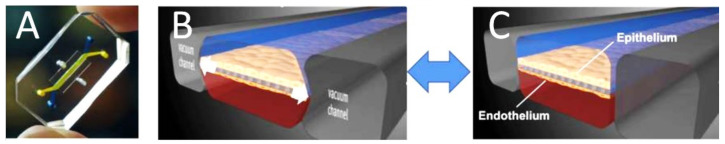
The first Organ-on-Chip. (**A**) The first model of Lung-on-Chip was entirely made of transparent silicon (PDMS) and consisted of two microfluidic chambers (blue and green) separated by a porous membrane (**B**,**C**) coated with human ECM and seeded with pulmonary epithelial (upper chamber) and endothelial (lower chamber) cells. Two lateral (vacuum) chambers were positioned along the main cell culture area to generate mechanical strains. Reproduced with permission [7].

The term “Organ-on-a-Chip” was first used in 2010 to describe the first “mechanically active” microfluidic model of the human lung [60]. The design of the Lung-on-a-Chip built on previous research conducted by Dan Hu et al. and published in 2007 concerning the establishment of a microfluidic model of the human airway [61]. Both the Lung-on-a-Chip and the previous airway model consisted of two parallel microfluidic chambers separated by a porous membrane coated with a thin layer of extracellular matrix, and lined with human lung epithelial cells (top chamber) juxtaposed to human endothelial cells positioned in the bottom chamber (Figure 2). While the airway model was built using a porous membrane made of rigid plastic (PET), the Lung-on-a-Chip incorporated an elastic membrane made of PDMS and held in between two laterals microfluidic chambers named “vacuum chambers”. The Lung-on-a-Chip was the first micro engineered in vitro system designed for simultaneously capturing the cellular, biochemical, and biomechanical determinants of TME of a human organ, including cell-cell and cell-matrix interaction, vascular shear stress and mechanical strain; all functional elements of the alveolar tissue. Ultimately, the combined use of an elastic membrane and vacuum channels resulted in a unique design that enabled the modelling of breathing motion (stretching), air flow and liquid flow within the same microdevice. The Lung-on-a-Chip [62] represents a milestone in the history of the in vitro modelling of human tissue-physiology. The remarkable success of the design of the first Organ-on-Chip is evident by the growing number of models that are built on the same concept, including gut [63], liver [64] and blood–brain barrier [65].

## 4. Synergistic Partnerships between Industry and Regulatory Bodies Are Driving Broad Adoption of OOCs and MPSs

The establishment of various consortiums and partnerships, particularly with regulatory bodies and pharmaceutical industries, has been essential in MPS development and adoption of the technology by pharmaceutical industries. Some of the key milestones are discussed in this section.

In 2011, the U.S. National Institute of Health (NIH) established the National Centre for Advancing Translation Sciences (NCATS) in response to the high failure rates of the drug development process. Then in 2012, the NIH MPS Program was founded as a collaborated effort of the NIH, the Defence Advanced Research Projects Agency (DARPA), and the FDA to accelerate the development of human MPSs for toxicology and drug development testing. The NIH MPS Program began with funding and providing advice to 19 investigators through two- and five-year collaborative agreements to create and integrate MPSs with human primary or stem cells that are sustainable and functionally represent 10 major organ systems [66]. The NIH MPS Program also established Tissue Chip Testing Centres to independently validate chips developed under the program. The International Consortium for Innovation and Quality in Pharmaceutical Development (also known as the IQ Consortium) has helped define MPS context of use within the industrial sector with the development of the IQ MPS Affiliate, which allows for cross-pharma collaboration, data sharing, implementation, and qualification of MPSs. The IQ MPS Affiliate has also pointed out the need for standards, comparing pre-clinical animal study results with MPS data, producing enough data for reliability and confidence in the technology, and increased throughput for MPSs to continue to grow and be adopted [67].

While the recipients of the original NIH MPS Programs were mainly awarded to investigators at various universities, a number of MPS companies have been created in the last decade such as TissUse GmbH (Berlin, Germany), Emulate, Inc. (Boston, MA, USA), MIMETAS Inc. (Leiden, The Netherlands), and Hesperos Inc (Orlando, FL, USA). Such companies have received the support of consortiums, initiatives, and regulatory bodies for the development of their systems and for using the technology in drug discovery which has helped with beginning to address the adoption needs pointed out by the IQ MPS Affiliate.

TissUse GmbH, based in Berlin, Germany, offers a proprietary, patent-protected “human-on-a-chip” platform which can be applied to a broad field of applications such as skin, vasculature, liver, brain, lungs, and lymph node. In 2017, TissUse GmbH announced they were a partner in the interdisciplinary consortium HiPSTAR (Human iPS Cell-based Blood Brain Barrier Technology in Alzheimer Research) which focuses on identifying the molecular mechanisms that lead to Alzheimer’s and developing therapies for the disease. The HiPSTAR consortium is coordinated by the University of Würtburg and is funded by the German Ministry for Education and Research. In terms of pharmaceutical partnerships, TissUse and AstraZeneca collaborated to create an MPS to study pancreatic islet-liver cross talking [68], which led to further collaboration to develop the model into a type 2 diabetes chip model. The company has also collaborated with Roche and Bayer to develop applications for their PMSs in drug research and risk assessment. In 2019, TissUse was certified under ISO EN 9001-2015, which certifies quality management in regard to products and customer satisfaction. Most recently in 2021, TissUse announced a signed collaboration agreement with Phillip Morris International to develop a human aerosol test platform.

Emulate, Inc. spun out from the Wyss Institute for Biologically Inspired Engineering at Harvard University and has since successfully scaled up the production of PDMS-based OOC devices, Bio-Kits, including human cells, and software to support their brain, colon intestine, duodenum, kidney, liver, and lung Chips. Emulate reports having “18 of the top 20 pharmaceutical companies as customers, including leading academic and government entities around the globe”; their research partners include Roche, Johnson & Johnson, Genentech, and Gilead Sciences and the U.S. Food and Drug Administration (FDA). In April 2017, Emulate, Inc. announced a multi-year Cooperation Research and Development Agreement (CRADA) with the FDA to use their OOC models for toxicology testing with their Liver-Chip, which is reported to have been completed successfully; they found the Liver-Chip model had high specificity and sensitivity, good power, and low variability [69], which helped in furthering the regulatory acceptance of OOC models. More recently, in October 2020, Emulate announced another CRADA with the FDA to evaluate the efficacy of the COVID-19 vaccines and to understand the immune response to SARS-CoV-2.

MIMETAS Inc. was originally founded in 2013 in Leiden, the Netherlands and opened a branch in the U.S. the same year. The company’s main technology is the OrganoPlate^®^ which is a microfluidic 3D culture plate that supports up to 96 tissue models in a single plate for high throughput screening. In 2018, the company opened a subsidiary in Tokyo, Japan. The company reports “working with nearly all major pharmaceutical companies and many top-tier academic institutes”. In 2021, MIMETAS Inc. and some of their collaborators, including Roche Innovation Centre, Pfizer, GlaxoSmithKline, the University of Applied Sciences, north-western Switzerland, and the Swiss Centre for Applied Human Toxicology, published their research for a renal tubule-on-a-chip for detecting drug-induced toxicology [70].

Hesperos Inc. claims the title “the original human-on-a-chip company” with CEO Michel Shuler coining the model “animal-on-a-chip” which would later become “OOC” in the early 1990s. Michel Shuler and Chief Scientist James J Hickman were among the original grant awardees from the NIH MPS Program, though the company was not formally established until 2015. Hesperos’s MPS technology has since been the recipient of various NIH grants to support developing their technology, and Hesperos has partnered with various pharmaceutical companies and universities such as AstraZeneca, Roche Pharmaceuticals, and the University of Central Florida in developing MPS, such as a neuromuscular joint (NMJ) system for personalized amyotrophic lateral sclerosis (ALS) [71], and a multi-organ system to evaluate anticancer therapies [72].

The 2021 Advancing Regulatory Science at FDA Focus Areas of Regulatory Science (or FARS) report mentions the FDA is working to replace, reduce, and refine animal studies by developing new models to evaluate and predict the safety and efficacy of FDA regulated products; MPSs are one of the examples listed as an alternative of interest. As MPS and OOC models continue to be developed and become more prevalent in research, the need to assess the performance capabilities of the models for safety and efficacy testing is important for laying out a regulatory roadmap for such systems. While MPS systems have been used for preclinical safety testing and internal decision making within the pharmaceutical industry, MPS assays have not been used in regulatory documentation for drug approval. In order for MPSs to take the next step from an investigative research device to a validated drug development tool, it will be imperative to have adequate validation and understanding of the systems to ensure they satisfy regulatory review requirements [73].

Shortages in nonhuman primates, in part due to COVID-19 vaccine testing and the continued ethical concerns, highlights the need for alternatives to animal models for drug and vaccine development. The CRADA between Emulate and the FDA to develop a system to understand COVID-19 and efficacy of its vaccines is a recent example of how regulatory bodies are turning towards MPS development for current medical issues; MPSs have the potential to help with the rapid development of new therapies without the need of animals, which would be helpful for future potential pandemics. As MPSs continue to evolve, it will be important for research, industry, and regulatory bodies to work together to support the development and widespread use of these systems to allow this technology to be used at its full potential.

## 5. Prominent Chip-Designs and Evolution of Technological Trajectories

**Figure 3:**
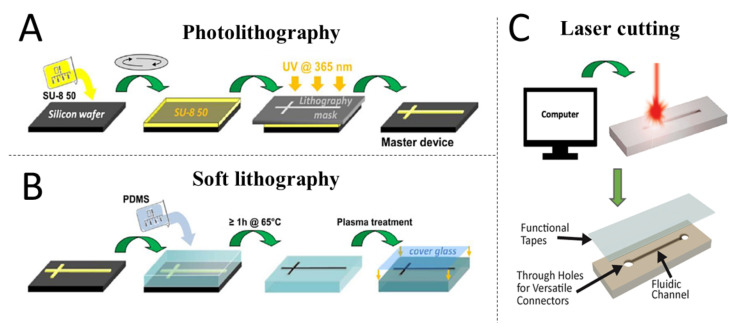
Typical fabrication techniques used for generating microfluidic constructs for Organs-on-Chips. Schematics capturing the main steps in (**A**) photolithography, (**B**) soft lithography and (**C**) laser cutting. Reproduced with permission from [74] and under Creative Commons license from [75].

In the past 20 years, the combined use of soft lithography (Table 1 and Figure 3), photolithography and PDMS resulted in an enabling microfabrication strategy that allowed a growing number of researchers to generate, in a relative short time-period, a variety of OOC models with demonstrated tissue and organ-level function. Under the increasing demand for more complex and predictive in vitro models for drug testing, the field of OOCs has rapidly evolved to incorporating multiple tissue-specific cells including stromal, microvascular endothelial and immune cells. Despite the different fabrication strategies, the use of parallel microfluidic chambers, frequently made of PDSM, remains the most prominent design feature across nearly all existing OOCs and MPSs. Currently, a shift in the paradigm is in progress as some of the most recent publications suggest that the field is moving away from the concept of confining cells in parallel microfluidic chambers made of PDMS (or other plastics) as hydrogels and other bio-inspired materials are taking the place of traditional synthetic polymers, a transition often enabled by 3D printing.

In this section of the review, we will trace the design evolution of vascularized OOCs using the Lung-on-Chip and the Brain-on-Chip as two examples.

### 5.1. Tracing the Design Evolution of the Lung-on-Chip

**Figure 4:**
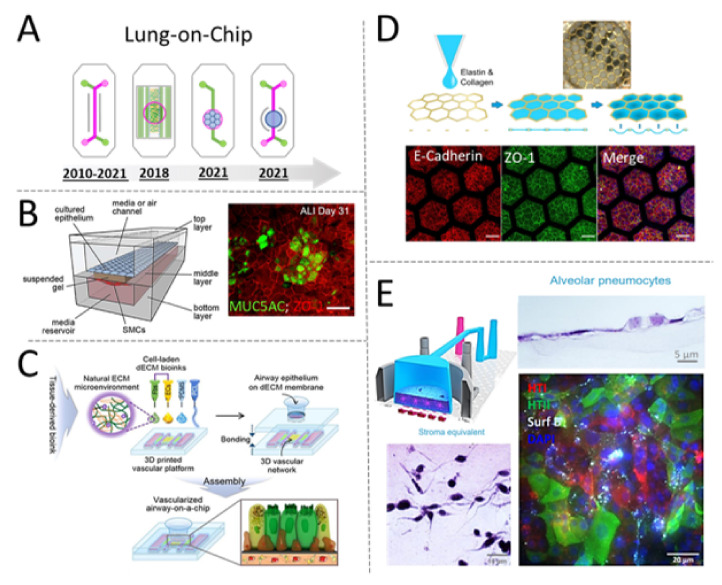
Compartmentalized microdevices used for modelling the human lung on-Chip. (**A**) Schematic overview of Chip-designs used over time for building microfluidic models of the human lung. Since the development of the first model of Lung-on-Chip, several research groups have used a sandwich-Chip design consisting of two microfluidic compartments (green and pink) separated by a porous membrane frequently made of PDMS or PET to micro engineering different parts of the human lung, including the alveolar-capillary interface and the airway mucosa [82,83]. (**B**) In 2018, Dr. Humayun and colleagues reported the fabrication of a model of Airway-on-Chip integrating smooth muscle cells (SMC) encapsulated within a “suspended gel” positioned in between the epithelial and endothelial compartment. Epithelial cells (Calu-3) growing for over one month in this triple-coculture system expressed various tissue-specific markers, including MUC5AC and ZO-1 when at air–liquid interface. Reproduced with permission [85]. (**C**) In 2018, Dr. Ju Young Park et al., published the first bioprinted model of a human airway. The system consisted of a bioprinted micro-vascularized construct made of tissue-derived ECM and fibrin. An open-top construct made of PDMS was layered on top the bioprinted vascularized-construct and seeded with human airway epithelia cells, grown to form a polarized epithelial monolayer showing typical cell-structure and biomarkers of a healthy airway. Reproduced with permission [86]. (**D**) In 2021, Dr. Zamprogno and colleagues demonstrated the possibility of generating a micropatterned and stretchable porous membrane using a thin gold mesh (with a pore size of 260 µm) embedded in elastin and collagen as a scaffold to supporting an array of 40 alveoli. Alveolar epithelia cells growing within this stretchable membrane grown to form a tight monolayer lined with junctional proteins (E-Cadherin, ZO-1) and expressing other markers of mature pneumocytes. Reproduced under Creative Commons license from [88]. (**E**) In 2021, Dr. Varone et al., developed an open-top model of an alveolar Lung-on-Chip incorporating pulmonary stromal cells (lung fibroblasts) into a stretchable and micro-patternable hydrogel. Primary human pneumocytes growing at air-liquid interface on the apical surface of the hydrogel were shown to grow and acquire typical markers of healthy alveolar cells, including typical cell morphology and expression of type I and II cell markers, including surfactant. Reproduced with permission [87].

Since the development of the first model of Lung-on-Chip, several research groups have used a “sandwich-Chip” design consisting of two microfluidic compartments (green and pink) separated by a porous membrane (frequently made of PDMS or PET) to microengineering different parts of the human lung, including the alveolar-capillary interface and the airway mucosa [82,83] (Figure 4A).

The original concept of Lung-on-Chip was conceived for recapitulating the architecture of the alveolar-capillary interface consisting of the juxtaposition of epithelial and endothelial cells. However, the tiny microfluidic chambers were not suited for the incorporation of a stromal compartment. As described in the previous section of this review, hydrogels and biocompatible scaffolds have been used for over a century to provide cells with a biomimetic microenvironment that classic 2D methods cannot match. Three-dimensional (3D) cell culture is particularly relevant for culturing mesenchymal cells, such as fibroblasts and other tissue-specific stromal cells that play a central role in maintaining tissue homeostasis via regulation of important cellular cell mechanisms, including cell replication and modulation of inflammatory responses [84]. Incorporation of these additional cell types in an OOC is necessary to generate better predictive systems capable of integrating multicellular responses to inflammatory molecules or drug-compounds targeting stromal or immune cells. Researchers working in academia and industry have adopted multiple strategies to expand the capabilities of OOC, frequently by adapting the microfluidic compartment geometries to accommodate cell-laden hydrogels (Figure 4B) or other biocompatible scaffolds [85].

Bioprinting and 3D-printing methods have recently emerged as attractive alternatives to traditional microfabrication techniques. One recent example of biofabricated MPSs comes from the research team lead by Prof. Dong-Woo Cho, who have successfully generated a fully bioprinted model of an airway [86]. The system integrates human endothelial cells and fibroblasts embedded in decellularized extracellular matrix isolated from pig trachea. Airway epithelial cells seeded on top of the bioprinted stromal compartment are accessible through an open-top chamber (Figure 4C). The tissue-specific ECM provides physiologically relevant cues that are critical to instruct the endothelial cells to form a 3D network resembling the natural architecture of vascularized tissues. The authors demonstrated the possibility of mimicking the allergic reaction to house dust mites. These allergens stimulate the expression of adhesion molecules and other vascular mediators of circulating immune cell recruitment into the inflamed tissue. Although still in its infancy, this model incorporates all the elements to become, in the future, an enabling human-relevant system for the testing of anti-allergenic compounds targeting the stromal and/or the immune component of an allergic reaction.

The use of hydrogels has resulted in an enabling strategy to better mimic composition and mechanical properties of the lung tissue. In the early models of Lung-on-Chip, epithelial and endothelial cells were seeded on a thin layer of ECM and, therefore, grown on a stiff (not physiological) and planar substrate. Recently, Dr. Zamprogno and colleagues demonstrated the possibility of generating a micropatterned and stretchable porous membrane using a thin gold mesh embedded in a mesh of tissue-specific proteins (elastin and collagen) (Figure 4D). The result was a stretchable and micropatterned surface where alveolar cells can grow into alveolar sac-like structures to form a tight monolayer lined with junctional proteins, expressing other markers of mature pneumocytes.

Using a different approach, Varone et al. [87] have recently demonstrated the possibility of generating a model of Lung-on-Chip including primary alveolar cells seeded on a patternable hydrogel laden with pulmonary fibroblasts (Figure 4E). One peculiar aspect of this updated Lung-on-Chip design is the presence of an openable chamber (open-top) designed to host the stromal compartment, consisting mostly of collagen I and human fibroblasts further lined with epithelial cells exposed to air (on top) and endothelial cells on the bottom channel. This Chip, therefore, is designed to provide direct access to the apical surface of the cell culture and to facilitate the introduction of air or potential drug compounds. Interestingly, the same design was shown to be compatible with the culturing of other epithelial cells and to enable the fabrication of multiple vascularized organ-models, including skin. Future development of this model could be extremely valuable to address the current lack of human-relevant models of lung fibrosis or cosmetics drug testing.

In summary, the design of the Lung-on-Chip appears to evolve to incorporate new elements such as patternable hydrogels laden with stromal cells and accessible through an open-top design. It is likely that the same design elements could be broadly incorporated into other epithelial models. For example, another elegant example of open-top design comes from the laboratory of Dr. Hu (the leading author of the publication describing the first model of Lung-on-Chip) who has recently developed the first model of a “blinking” Eye-on-Chip [89]. The system is designed to recapitulate the structural complexity of the ocular surface and it incorporates, for the first time, human cells derived from the cornea and conjunctiva in dome-shaped and micropatterned scaffold positioned in an open-top compartment interfaced with 3D printed eyelids. Electromechanical actuators control the blinking of the eye lids to simulate spontaneous eye blinking and generating physiologically relevant shear stress. This model, which was also shown to recapitulate some key aspects of an ocular disease known as “dry eye disease”, may serve as a future platform to study human-relevant ocular physiology and pathology for biomedical, pharmaceutical, and environmental applications. Given their demonstrated ability of mimicking human tissue structures and inflammatory responses, these recently developed models of the skin and eyes could be used in the near future as human-relevant alternatives to animals, such as rabbits, in the eye and skin irritation test [90].

### 5.2. Tracing the Design Evolution of the Blood-Brain Barrier-on-Chip

Neurovascular inflammation, initiated by vascular or genetic predisposing factors and aging [91], is a common denominator and trigger of a variety of pathologies of the central nervous system (CNS). Animal models and conventional 2D cell culture systems do not offer adequate solutions for deciphering the crosstalk between the cellular components of the human brain and circulating blood cells [92,93], which is ultimately central to the identification of new therapeutic targets for a variety of neurological disorders. Vascularized in vitro models of the human brain could help develop a better understating of the cellular mechanisms of neurological diseases and provide a testing-platform for identifying novel therapies. Here, we will review some of the main design concepts and applications of the Brain-on-Chip.

### 5.3. Endothelialized Sandwich Chips

**Figure 5:**
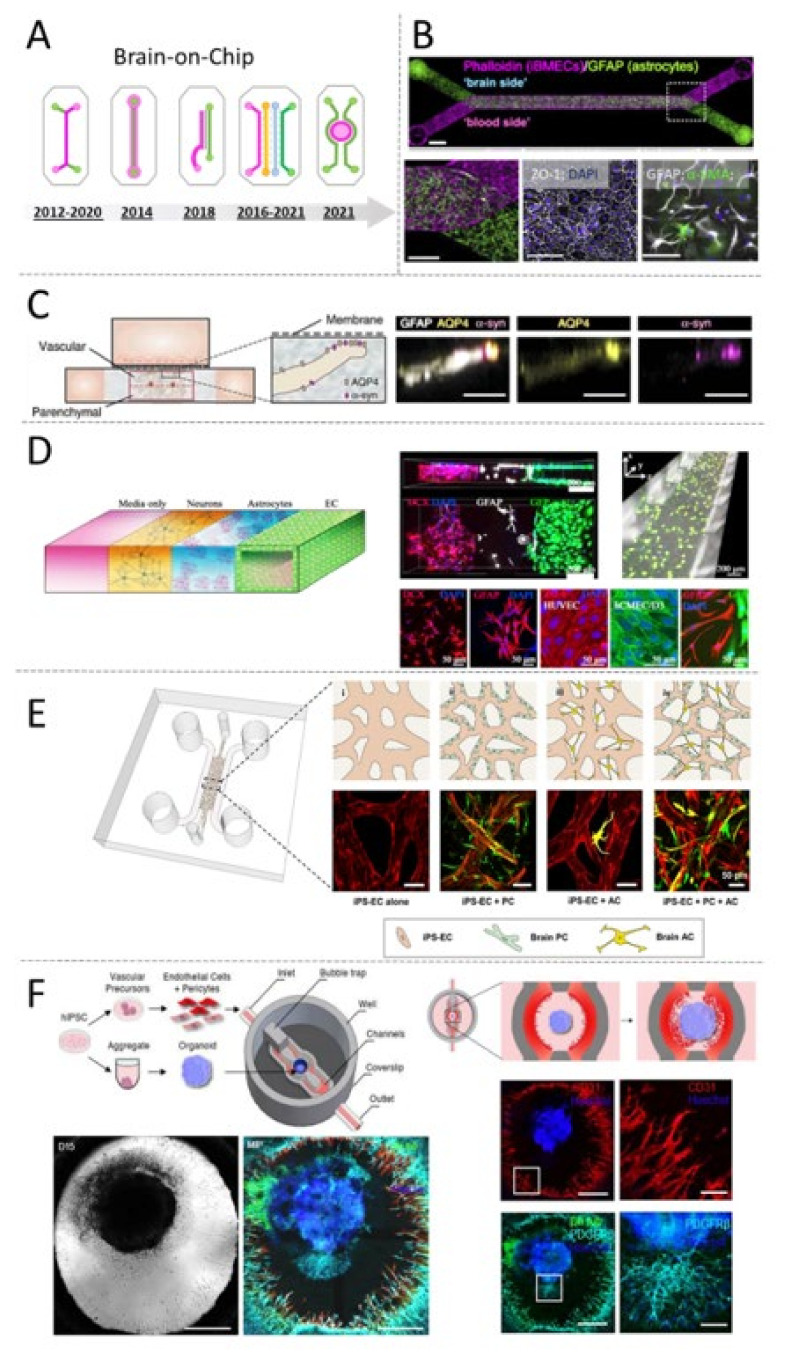
Compartmentalized microdevices used for modelling the human blood-brain-barrier on-Chip. (**A**) Schematic overview of Chip-designs used over time for building microfluidic models of the human blood-brain barrier (BBB). Several groups used a sandwich-Chip design that, similar to the “first” organ-on-chip, consists of two microfluidic compartments (green and pink) separated by a porous membrane frequently made of PDMS or PET [102]. In 2014 Dr. Tourovskaia et al. [103] reported the successful establishment of a vascularized model of BBB using a single-channel chip. Researchers were able to generate a hollow and perfusable vascular compartment (green) into a collagen hydrogel (pink) containing living perycites. A glass rod was used to stabilize the perfusable structure during hydrogel polymerization. Notably, a similar structure was obtained by Herland et al. in 2016 using a sandwich-Chip design [104]. In this case the team adopted a “viscous fingering” approach to create a luminal vascular compartment. In 2018, Dr. Wevers et al. [105], demonstrated the utility of a high-throughput plate-system integrating up to 96 chips for drug screening purposes. Each individual Chip consists of two parallel microfluidic chambers separated by “phase guides” (grey). One of the two chambers (pink) is filled up with a cell-laden hydrogel containing parenchymal cells while the second channel contains only brain-endothelial cells. The Chip is designed to recreate the endothelial-brain tissue-tissue interface that is relevant for modelling the mechanisms of molecular transport across the human BBB. Interestingly, a similar tissue-structure was obtained by other groups using a sandwich-Chip design [106] (2020). This most recent design, however, was not conceived for high-through screening purposes. In 2017, Dr. Adriani et al. [107], reported a novel 3D model of neurovascular unit on-Chip consisting of four parallel microfluidic chambers separated by fenestrated walls (pillars, grey). The two central chambers were filled up with hydrogels laden with neurons (orange) or astrocytes (blue). One of the two lateral chambers (green) was seeded with brain endothelial cells to form a blood-vessel like structure while the 4th chamber (pink) was used to perfuse cell culture medium. In 2018, and later in 2021, a similar design was used to generate a micro-vascularized model of the BBB [108,109]. In 2021, Salmon et al. reported the first 3D printed model of vascularized brain-organoid consisting of a central round-chamber (pink, open-top) designed to hold an iPSc-derived brain organoid surrounded by two endothelialized microfluidic channels (green) [110]. (**B**) BBB-on-Chip model consisting in iPSc-derived brain cells including HBMECs and astrocytes seeded on the opposite sides of a porous membrane in a “sandwich-chip”. The porous membrane allows for limited cell-cell interaction; astrocytes interact with endothelial cells via GFAP-positive protrusions. Reproduced with permission from [98]. (**C**) Encapsulation of astrocytes into a collagen-hydrogel allows for the maintenance of a quiescent phenotype and enables the maturation of polarized end-feet-like structures. Reproduced under Creative Commons license from [106]. (**D**) Brain cells growing in 3D-hydrogels confined within parallel microfluidic chambers that allow for direct (membrane-free) cell–cell interaction. Reproduced with permission from [107]. (**E**) A recent example of micro-vascularized OOC model of the human BBB including iPSc-derived endothelial cells (EC), pericytes (PC) and astrocytes (AC). Reproduced under Creative Commons license from [109]. This most recent example leverages the ability of brain-cells to self-assemble into functional 3D structures that closely mimic the tissue-architecture and transport-function of brain micro vessels. (**F**) The first 3D-printed microfluidic model including a vascularized iPSc-derived brain-organoid [110].

Endothelialized Organ-on-Chip models of the brain, including the blood–brain barrier (BBB) and the neurovascular unit (NVU) are frequently built around the concept of compartmentalizing different cell-types into parallel microfluidic chambers made of PDMS [94] (Figure 5A). Microchambers are frequently separated by a porous membrane to form a “sandwich”. In these devices, endothelial cells and other tissue-specific cells such as neuronal cells (sometimes including glial cells and/or neuronal progenitors) are cultured on the opposite sides of the porous membrane to form the neuronal (or brain) compartment and the vascular (or blood) compartment. When compared to traditional static cell culture methods, the microfluidic nature of these devices allows cells to grow in a dynamic microenvironment that closely resembles the physiology of highly vascularized organs such as the human brain.

Building a predictive model of brain-on-chip requires the combination of multiple types of brain cells that are difficult to obtain and frequently not replicative (therefore hard to expand in vitro). To overcome this limitation, Kilic et. al. have co-cultured a brain cell-line (NTERA-2) capable of differentiating into astrocytes and neuronal progenitors, together with foetal-derived neuronal cell to reconstitute the heterogeneous brain TME in a scalable and user-friendly sandwich-chip [95]. To mimic the brain niche, the authors co-cultured the human teratocarcinoma line NTERA-2 and foetal neural progenitor cells (hNPCs) in the brain compartment, while the vascular compartment was seeded with immortalized human brain microvascular endothelial cells. Confocal imaging confirmed the formation of physiologically relevant markers of the human brain, including formation of neuronal clusters positive for markers of foetal brain, such as NF200 and MAP2ab. More importantly, the authors demonstrated the applicability of these devices for studying the functional crosstalk between the cellular elements of the brain-on-chips and for modelling of tissue-guided chemotaxis of neuronal progenitors, a cellular process that is central during brain regeneration after injury and that is extremely challenging to study in animals. Ultimately, this device provided a user-friendly platform to model key aspects of the CNS within a controlled microenvironment that allows hNT2 cells to differentiate simultaneously into neuronal-like and glial-like cells. Although the system lacks control of fluid-flow, the Chip-design allows for the facile generation of chemo-attractive gradients that are relevant for studying migratory cell dynamics. The lack of mature cellular structures such as synapses, however, limits the applicability of this system to the modelling of neuronal disorders.

Finding a reliable and physiologically relevant source of human brain cells has been the main limitation associated with the modelling of complex functions of the central nervous system for a long time. Only recently, the establishment of robust methods for generating stem-cell derived brain cells has allowed researchers to generate a relatively large number of brain cells, enabling the development of scalable in vitro models of the brain [96,97]. We have demonstrated the possibility of generating an isogenic model of the human BBB (Figure 5B) [98]. Induced pluripotent stem cells (iPSc) were used to derive astrocytes and cortical neurons, both seeded in the brain compartment, as well as brain microvascular endothelial cells seeded within the vascular compartment of a sandwich-chip. Astrocytes were shown to protrude through the porous membrane and form 3D structures connecting the brain and vascular compartment, reminiscent of the astrocytic end-feet of the NVU. Endothelial cells formed a compact monolayer expressing tight-junction proteins and tissue-specific molecular transporters that are all elements required to recapitulate the cerebrovascular tissue structure and function of the BBB. When exposed to physiological fluid–flow (shear rate ≈ 6 dyne/cm^2^), endothelial cells show increased expression of tissue-specific markers, indicating that the dynamic microenvironment of the BBB-on-Chip enhances the maturation of iPSc-derived endothelial cells. The vascular wall of this OOC system provided an effective barrier-function to the diffusion of large molecules present in the human blood, therefore, capturing one of the key physiological functions of the human BBB. When the vascular compartment of healthy (but not inflamed) chips was perfused with whole blood (using methods that we had previously described [99]), it maintained viability and functionality. As expected, Chips inflamed with TNF-alpha, demonstrated loss of barrier-function and vascular-to-brain leakage of large blood-borne factors (such as IgG), including neurotoxic substances naturally present in the serum of human blood. Of note, this model has shown, for the first time, the possibility of modelling genetically inherited and patient-specific disorders of the human BBB, thereby presenting a functional proof-of-concept that could boost the development of new OOCs models for precision medicine.

The combined use of Organ-on-Chip technology and iPSc-derived brain cells represents a new frontier in generating of physiologically relevant models of the human brain. Recent publications suggest that this technology could be applied, in the near future, for deciphering the altered cellular crosstalk occurring in the brain-tissue of patients affected by neurodegenerative diseases such as Parkinson and Alzheimer [100]. Other researchers are leveraging the reproducibility of iPSc-derived cells to establish physiologically relevant platforms for the rapid-screening of novel brain-targeted drug-delivery strategies [101].

### 5.4. Chips Incorporating Hydrogels

Three-dimensional (3D) culturing of brain cells has recently emerged as an important aspect of modelling of brain functions in vitro. For instance, growing primary human astrocytes and neurons in biocompatible scaffolds has resulted in enhanced glutamate metabolisms and neuronal firing [111,112], both cellular functions that are relevant to microengineering of physiological models of the human brain. By combining 3D-cell culture with microfluidic OOC, Ahn, S.I. and al [106] have previously reported a human BBB-on-Chip model inclusive of brain endothelial cells and pericytes, both growing on the top layer of a sandwich-chip, and astrocytes encapsulated within a 3D scaffold, located in the bottom compartment of the Chip (Figure 5C). Interestingly, the use of a scaffold composed only of Collagen I resulted in an enabling strategy to support the natural ability of astrocytes to self-assemble into a 3D network with polarized expression of aquaporin 4 (AQP4) and decreased reactive gliosis markers. The reduced reactive astrogliosis allows for the generation of more sensitive models of neuroinflammation, which could be used to better predict the response to extrinsic inflammatory molecules.

One drawback of the sandwich-chip design is that the porous membrane, separating the astrocytes from the endothelial cells, limits the tissue–tissue interaction, representing a major obstacle to the formation of mature end-feet structures. Adriani et al. [107] have developed a microfluidic model of the neurovascular unit using a membrane-free design that consists of four parallel chambers separated by trapezoidal structures named “pillars” (Figure 5D). Pillars are used to form fenestrated walls that provide structural support to hydrogels and, at the same time, enable the formation of cell-cell interactions within a 3D microenvironment. The authors used primary rat astrocytes and neurons together with human cerebral microvascular endothelial cells. Each cell type was cultured into a separated hydrogel, a strategy that permits each cell type to virtually grow in an optimized microenvironment as opposed to the sandwich design, where multiple cell types are co-cultured on the same compartment and must share the same substrate, including ECM composition and stiffness. The microdevice reported by Adriani et al., therefore, allowed for direct interaction of astrocytes with endothelial cells, although formation of end-feet was not reported. Moreover, the system was proven to be amenable for performing quantitative assessment of neuronal responses via imaging of neuronal firing, as well as detection of molecular transport of soluble factors through the vascular wall of the endothelialized compartment.

### 5.5. Micro-Vascularized Organ-on-Chips

Microengineering the vascular component of human organs in vitro has been a challenge of primary relevance for tissue-engineers. Although methods describing endothelial “tube formation” and capillary-like structures have been vastly described since the beginning of the 21st century [113], these constructs were not perfusable. Advances in microfluidic technologies in the past 20 years have been instrumental in addressing the long-standing challenge of engineering vascularized and perfusable micro-tissues [114] (Figure 5E). By harnessing the angiogenic mechanisms that govern the formation of new capillaries in living tissues, multiple groups have demonstrated the possibility of co-culturing human endothelial cells and fibroblasts (or other mesenchymal cells) to guide the generation of micro-vessel-like structures (≈10 in diameter) into biocompatible hydrogels, thereby breaking the previous impasse that hampered the engineering of a functional capillary bed in vitro.

The research lab of Prof. Kamm, who has pioneered the field of micro-vascularized OOCs, has previously developed a 3D microvascular model of the BBB inclusive of iPSc-derived endothelial cells, astrocytes and pericytes [108]. More recently, the team of Prof. Kamm has demonstrated the utility of this model for dissecting the dysfunctional crosstalk occurring at the neurovascular interface between brain astrocytes and the metastatic cancer cell line “MDA-231” [109]. Results of this work suggest a key role for the astrocyte-cancer cell crosstalk in guiding the extravasation of metastasis into the brain, indicating that cancer cell extravasation does not result in a significant decline in BBB barrier-function.

Potential improvements to this model may lead to the establishment of an immune-competent system that could potentially account for the well-known contribution of circulatory and resident immune cells in supporting tumour progression, including metastatic extravasation.

As for other bioengineered systems, the evolution of OOCs is closely connected to the development of novel fabrication methods as well as advancements in understanding of fundamental biologic principles regulating cell and tissue functions. The BBB-on-Chip appears to evolve to include stem-cell derived (mostly iPSc-derived) brain cells and, most recently, organoids in 3D hydrogels. The general trend is to build “bioinspired” rather than “biomimetic” systems designed to leverage the natural ability of animal cells to self-assemble into functional higher order tissue-structures such as microvascular capillaries or organoids.

In line with this trend, Salmon et al. [110] have recently demonstrated the possibility of 3D printing a developmentally inspired model of Brain-on-Chip. The 3D-printed framework of the device consists of a central open-top compartment designed to facilitate the seeding of brain organoids and surrounded by two microfluidic channels lined with a vascularized hydrogel (Figure 5F). In these devices, iPSc-derived brain endothelial cells, pericytes and organoids are initially cultured separately. However, since cells are all encapsulated in the same hydrogel, they can move following gradients of differentiating factors and chemotactic cues released by other cell types as they would do in vivo during brain organogenesis. This system could be used in the future for studying the fundamental principles that regulate the interaction of endothelial cells, pericytes and other brain cells to possibly gain a better understanding of cellular and molecular mechanisms underpinning the insurgence of neurodevelopmental disorders that are currently difficult to study in other models.

## 6. The Emergence of Bioprinted Microphysiological Systems

**Figure 6:**
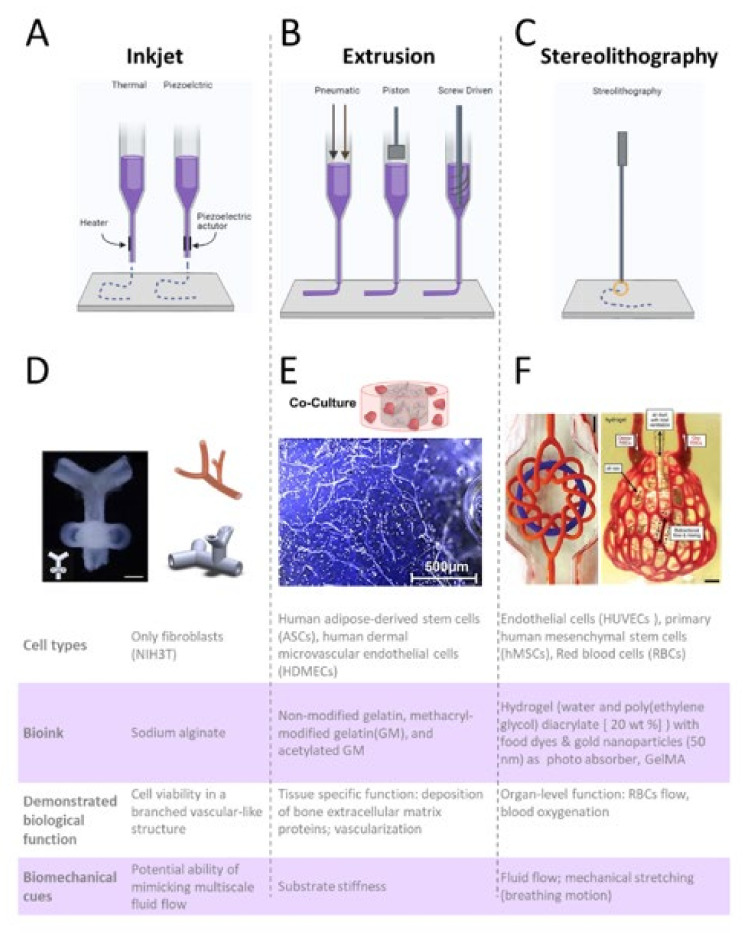
Bioprinting techniques used for generating of vascularized micro-tissues. (**A**) Process of Inkjet, (**B**) extrusion, and (**C**) stereolithography techniques are depicted at the top of the figure. Created with BioRender.com. One example of vascular construct is demonstrated for each technique, including: (**D**) a branched construct, reminiscence of vascular tree obtained via inkjet bioprinting of alginate droplets with or without fibroblast cells. Reproduced with permission from [119], (**E**) Vascular bone construct obtained via extrusion bioprinting of laden gelatin (modified and non-modified). Reproduced under Creative Commons license from [120], (**F**) 3D alveolar construct including of alveolar sacs and perfusable microchannels obtained via stereolithography. Reproduced with permission from [121]. The bottom panel of the figure summarizes the main biological and mechanical functions/properties of the construct.

The origins of bioprinting can be traced back to the early 2000s, when the research group of Thomas Bolan (at Clemson University) modified a commercial inkjet printer into a bioink printer (Figure 6A) capable of live-cell tissue printing via precise dispensing of single droplets of cell compatible material (bioink) [115]. At the beginning of the 21st century, bioprinters were only accessible to a limited number of researchers who worked in specialized bioengineering laboratories. Since then, through the diffusion of commercial bioprinters [116], a new wave of scientists gained access to the bioprinting technology. Researchers have tackled increasingly difficult problems, including the ability to vascularize thick micro-tissues obtained from the 3D bioprinting of multiple layers of cell-laden bioink. Bioprinting supports specific functionalities and biomaterial heterogeneity which could mimic the functionality of cells [117]. This enables the creation of a biomimetic microenvironment with heterogeneous 3D structures that cannot be obtained with traditional microfabrication methods.

The possibility of bioprinting 3D constructs that closely mimic the architecture and function of native tissues has been well documented in several publications [118], (Figure 6). Table 2 summarizes some of the best established and common methods, materials and parameters used for the bioprinting of MPSs. Advantages and disadvantages of each method are briefly summarized in Table 3. The following paragraphs of this review will discuss the prevailing bioprinting techniques and their most recent applications in the building of MPSs, with special emphasis on extrusion bioprinting and stereolithography (SLA).

**Table 2:** Methods, materials, and parameters frequently used for bioprinting of vascularized constructs and Microphysiological systems.

Bioprinting Technique	Biomaterials	Cell Type	Crosslinking	Rheological Properties	Printing Conditions	Ref.
**Extrusion**	GelMA PDMS	Liver HepG2/C3A	Photo Crosslinking (UV, 850 mW, 15s from 8.5 cm distance)	Elastic module of 5 kPa	Speed: 2 mm/s Pressure: not specified Temp: RT	[122]
**Extrusion**	GelMA	Human adipose tissue-derived mesenchymal stem cells (hAD-MSCs)	Photo Crosslinking (UV, 335 nm)	Shear rate of 0.01 to 1000 s^−1^ at 25 °C	Speed: 260 mm/min Pressure: 2.8 and 3.8 psi Temp: 30 °C or 37 °C	[123]
**Coaxial Extrusion**	GelMA with gold nanorods and alginate	Cardiac fibroblasts, cardiomyocytes	Photo Crosslinking (UV, 800 mW)	Elastic module of 4.2 ± 0.3 kPa (0.1 mg) and 4.7 ± 0.3 kPa (0.25 mg)	Speed: 1–6 mm/s Pressure: not specified Temp: RT	[124]
**Extrusion**	GelMA, Pluronic PDMS	Human colorectal cancer cells	Photocrosslinking (UV, 400 nm)	Not reported	Speed: 100 mm/min Pressure: 2–4 kPa Temp: 26 °C	[125]
**Extrusion**	Gelatin(G) GelMA(GM) Acetylated GelMA (GMA)	Human adipose-derived stem cells (ASCs), human dermal microvascular endothelial cells (HDMECs)	Photo crosslinking (UV, 365 nm, 0.54 J cm −2, 60 sec)	Storage Modulus: (G) 556.20 Pa (GM) 1373.21 Pa (GMA) 500.25 Pa	Speed: 0.22–0.3 μL/s Pressure: not specified Temp: 21–22 °C	[120]
**Stereolithography (SLA)**	PEGDA GelMA	Primary human mesenchymal cells, Red blood cells (RBCs), HUVECs, hepatocytes	Photocrosslinking (light, 405 nm, 16.4 mW/cm2, 6–40 s to 120 s)	Elastic modulus of PEGDA::10 % strain, 24 kPa	Speed: ≤12ml/hr Resolution: voxels of 250 pL Temp: RT	[121]
**Stereolithography (SLA)**	GelMA with PEGDA	Human bone marrow mesenchymal cells	Photocrosslinking (UV, 355 nm, 45 s)	Elastic modulus: 40% strain	Speed: 2 mm/s Temp: RT	[126]
**Stereolithography (SLA)**	GelMA PEG-bis-(acryloyloxy acetate)	Immortalized hepatocytes (HepaRG) and human stellate cells	Photocrosslinking(blue light illuminator for 30 s/layer)	Not reported	Not specified	[127]

**Table 3:** Overview of advantages and disadvantages of different bioprinting methods.

Bioprinting Technique	Advantages	Disadvantages	Ref.
**Inkjet bioprinting**	High throughput, speed, precision, reproducibility, wide availability	Thermal and mechanical stresses by Thermal droplet formation, possibility of cell membrane damage due to working frequencies of 15–25kHz in acoustic droplet formation.	[117]
**Extrusion bioprinting**	Broad range of material, economic, universality, versatile, allow customization	Resolution limited to 100μm, only 40% to 80% cell viability, unsuitable for complicated network structure	[116], [128], [129]
**Stereolithography** **(SLA)**	Higher resolution, cell viability up to 90%, suitable for multiscale network cannels	Expensive, limited number of biocompatible resins, chances of higher level of cytotoxicity	[116], [130], [117]
**Digital Light Processing** **(DLP)**	Constant high printing speed regardless of complexity of structure, excellent mechanical property and structural integrity, printing accuracy	Expensive resins material; Low mechanical property: risk of crack or deteriorating over time	[131], [132]
**Laser assisted bioprinting** **(LAB)**	High resolution (varies from pico- to micro-scale, printing speed, high cell activity, precision	Costly due to use of laser source	[116], [133]

### 6.1. Extrusion Bioprinting

Extrusion bioprinting uses a mechanically driven system (Figure 6B; pneumatic, piston-driven, and screw-driven) to expel filaments of bioink through a nozzle. The controlled and sequential deposition (layer-by-layer) of filaments [131] results in the generation of a 3D construct of variable dimensions and heterogeneous composition. Our current knowledge in cell biology, including molecular constituent of TME and ECM, are based on over a century of in vitro studies. Hydrogel-forming proteins have been extensively characterized and adapted for the 3D culture of several cell types. Unfortunately, most of these hydrogels are not “shear thinning” and cannot be used for the direct biopriting of 3D micro-tissues.

Because of their demonstrated ability to support cell viability and function in vitro, hydrogels forming proteins such as collagen I or the laminin-rich hydrogel Matrigel^®^ are considered as gold-standard biomaterials for 3D cell culture. However, these molecules are not printable at room temperature or 37 °C. During the extrusion process, the bioink needs to be in the liquid phase to avoid nozzle clogging, but viscous enough to adhere to the substrate (support) and hold the printed shape. Printing resolution and fidelity depend mostly on the rheological properties of the bioink, including viscosity and storage modulus. The viscosity is a measure of the natural fluid (bioink) resistance to flow, while the storage modulus is a measure of the energy stored in the elastic structure of the bioink.

While ideal for generating 3D scaffolds of high printing resolution, bioinks with high viscosity are not suitable for the extrusion of living cells, given the high shear stress, certainly caused during extrusion of highly viscous bioinks which could damage the cell membranes, with obvious negative impact on cell viability [134]. Generally, a bioink is considered bioprintable when the viscosity of the fluid decreases under shear (shear thinning).

More specifically, a storage modulus in the order of 10^2^–10^3^ Pa and viscosity comprised in the range of 30 to 6 × 10^7^ mPa s are considered suitable for extrusion [135]. For example, a solution containing 15% *w*/*v* gelatin (a denatured form of collagen) has a storage modulus of ∼30 Pa and viscosity of ∼50 Pa s [136]. Precise extrusion of gelatin is, therefore, not possible. Matrigel^®^, on the other end, maintains its liquid state only at low temperatures (below ∼12 °C) and it becomes a gel when at room temperature.

While not suitable for 3D printing, gelatin and other natural peptides forming hydrogels are frequently used in combination with biocompatible substances, often polymers (either natural or synthetic), for generating multicomponent-based bioinks. Alginate, Gelatin and Gelatin Methacrylate (GelMA) have been recently identified as the top three biomaterials most frequently combined to obtain printable bioinks [137].

Alginate is a polysaccharide obtained from brown algae (Phaeophyceae), normally present on the cell-walls of these organisms and frequently combined with other ECM molecules to obtain printable bioinks. Following ionic crosslinking with calcium, the polysaccharidic frame of alginate turns into an organized structure that resembles the molecular configuration of animal ECM [138]. Given its high biocompatibility, lack of immunogenicity and tuneable viscoelastic properties, alginate is the most widely used material for in biopriting. Human cells, however, do not adhere to alginate. The molecular backbone of the alginate, in fact, lacks the biological cues necessary for cell adhesion, proliferation, and function. Therefore, alginate is often combined with gelatin, or other bioactive substances to generate a 3D scaffold resembling the cell-adhesive properties and porosity of the native ECM.

Gelatin, a biomaterial obtained from the hydrolysis of collagen, has been extensively used in pharmaceutical and medical applications because of its high biocompatibility, biodegradability, and low immunogenicity. Its mechanical tenability and biofunctionality make gelatin-based hydrogels suitable to mimic the natural composition and stiffness of animal tissues [139]. While gelatin solution are not shear-thinning fluids per se, the modulus and viscosity can be adjusted to obtain extrudable bioinks via the addition of biological substances such as alginate or cellulose as well as via the chemical modifications of gelatin molecules [140,141].

GelMA is a chemically modified form of gelatin decorated with methacrylate groups. This semi synthetic hydrogel is optically crosslinkable using UV light (365–405 nm) when combined with a photoinitiator such as Lithium acylphosphinate (LPA) or Irgacure. Photocrosslinking facilitates the fabrication of sTable 3D constructs [123]; however, this process can be toxic to cells. Optimization of parameters such as light intensity and time of exposure as well as concentration of the photoinitiators themselves, is critical to limit the formation of free radical species and eventual cell damage and to control the stiffness of the crosslinked construct. Hydrogels made of GelMA have been frequently used to generate the cell laden, perfusable microengineered system with the different cell types [142].

The use of biological polymers obtained from living organisms has been instrumental for bioprinting 3D scaffolds designed to provide a favourable microenvironment for cell survival and function in vitro. Because of their animal-origin, however, natural hydrogels are subjected to significant batch-to-batch variability. Additionally, a major hurdle in the clinical translation and integration of biomaterials is their non-compatibility with current good manufacturing practices (cGMPs) which is also a barrier for the use of animal-derived molecules in a clinical setting [143].

The emergence of synthetic polymers, used alone or in combination with natural biomolecules to obtain semi-synthetic bioinks, could reduce batch-to-batch variability and improve experimental reproducibility [128]. Among others, polyethylene glycol (PEG) and its derivatives PEG diacrylate (PEGDA) and PEG methacrylate (PEGMA), are the synthetic materials most frequently used in bioprinting. These polymers, generated by the polymerization of ethylene oxide, can be used to form a highly biocompatible, hydrophilic, and porous scaffold that facilitates the exchange of cell nutrients and hormones. PEG and its derived materials (similarly to GelMA) allow hydrogels to be photo-crosslinked via UV lights. However, PEG lacks the biological cues necessary for cell adhesion (likely alginate), therefore, it must be combined with collagen, gelatin, or other bioactive substances to generate a biocompatible semi-synthetic scaffold. Because synthetic biomaterials are produced in a tightly controlled setting, these materials allow for higher reproducibility and are more suitable for generating 3D-printed constructs compatible with cGMPs. It is anticipated that in the near future, synthetic and semisynthetic bioinks will replace natural hydrogels, further decreasing the use of animals in research.

### 6.2. Limitations of Extrusion Bioprinting

Extrusion bioprinting is versatile and allows customization through multiple print heads and nozzles to generate complex structures with high overall porosity [103,135,136,137,138]. The technique can deal with higher ranges of viscosity in comparison to previous inkjet printing systems, however, there is some glaring limitations. For example, the excessive shear force generated during the extrusion of viscous bioinks can have a negative impact on cell viability, which typically ranges between 40–80%. In extrusion biopriting, it critical to use optimized bioinks that must be formulated to provide the biochemical cues necessary for cell adhesion and survival, as well as optimal rheological properties compatible with printing parameters such as nozzle dimension, extrusion pressure, speed and temperature [144]. Even in optimal conditions, however, extrusion biopriting is limited to producing constructs with a resolution of ≈100 μm [116], which is a major constraint to the modelling of hierarchical microvascular networks [145].

### 6.3. Examples of MPSs Obtained via Extrusion Bioprinting

One recent example of MPS obtained via extrusion bioprinting includes the work of Yi et al. who have developed a patient derived glioblastoma (GBM)-on-chip model incorporating vascular endothelial cells and patient-derived brain tumour cells [146]. The authors demonstrated that the 3D bioprinted structure can be used to recapitulate the structural, biophysical and biochemical properties of the native brain tumour, including an oxygen gradient, which is central to the modelling of the anaerobic tumoral niche in GBM. In order to print the GBM-on-chip model, three different types of bioinks were produced: a glioblastoma cell loaded hydrogel, a HUVEC loaded hydrogel and a printable silicone-mesh that worked as a scaffold for the bionks. The cell-laden bioinks were prepared by incorporating GBM cells or endothelial cells (HUVECs) in a solution made of brain-decellularized ECM (derived from market pigs) or Collagen I (10 mg ml^−1^). Both of the hydrogels showed cell viability greater than 90% but lower cell proliferation and angiogenesis when compared to the native (not bioprinted) brain decellularized ECM used as reference, suggesting that shear stress produced during extrusion in these hydrogels may cause limited cell damage. Ultimately, this work has shown the possibility of generating a GBM-on-chip model using biopriting of tissue-derived ECM, opening new perspectives on the possible use of native ECM for generating physiologically relevant MPSs.

A 3D model of liver-on-chip was recently obtained via bioprinting a cell-laden hydrogel made of GelMA and hepatic spheroids, obtained from the immortalized human HepG2/C3A line into a prefabricated bioreactor [122]. To determine the model-stability over time, the authors demonstrated that this hepatic construct remained functional for over 30 days, as assessed by monitoring the secretion of tissue-specific biomarkers such as albumin, alpha-1antitrypsin and transferrin, as well as via immunostaining for the other hepatocyte markers. The ability of this model to capture drug-induced toxicity was assessed via perfusion of acetaminophen, which resulted in an overall reduction of biomarker secretion, further accompanied by a significant reduction in cell viability and metabolic activity. This simple microfluidic model incorporating bioprinted hepatic spheroids, therefore, shows the potential for high throughput drug testing and assessment of hepatotoxicity.

### 6.4. Stereolithography

SLA, a rapid prototyping process developed in the late 1980s, is a light-base structuring technique that leverages the photosensitive chemistry of specific polymers to obtain 3D structures, with elevated speed and spatial resolution. SLA-based bioprinting frequently uses UV light to cure photosensitive bionks and hydrogels such as GelMA or PEG [147], which are combined with biocompatible photo-initiators such as LAP or Irgacure that are needed to mediate the photopolymerization of the bioink. Similar to extrusion bioprinting, SLA bioprinting fabricates 3D tissue constructs in a layer-by-layer manner (Figure 6C). The induction of photo-polymerization in SLA-based bioprinting is mainly controlled by light intensity, irradiation time and the concentration of the photoinitiator [9]. The light source can be controlled in multiple manners, including beaming-scanning or mask-image-projection [129]. In the first instance, the continuous drawing and solidification of a flat layer of bioink is achieved through the selective scanning of a focused laser beam [148]. After the curing of one layer, a second layer is added on top and the process of beaming–scanning starts again [149]. The mask–image–projection process employs a digital light processing technique (DLP) to create a defined mask image, which is efficient in the solidification of one entire layer of bioink by a single projection of the pattern image, resulting in the rapid curing of a layer of bioink [130]. These light-based structuring technologies allow for generating constructs with a resolution that is far higher than typical extrusion techniques [136,138].

### 6.5. Limitations of Stereolithography

Overall, the elevated printing speed and high resolution of SLA-based bioprinting offer some remarkable advantages for the generation [116] of intricate microfluidic channels of capillary diameters (≥ 50 um), however, there are a few drawbacks. When compared to extrusion-based bioprinting, SLA may result in increased levels of cytotoxicity due to the release of radical species during photo-crosslinking. The polymerized photo resins used for stereolithography have generally poor mechanical properties [9,137]. Additionally, SLA biopriting is typically limited to the use of one biomaterial at a time. The resulting construct, therefore, is generally made of one single material which does not reflect the heterogeneous nature of a native TME, ultimately relevant to modelling the tissue microenvironment that orchestrates the synchronous activity of multiple cell types and organ-functions.

### 6.6. Examples of MPS Obtained via Stereolithography

Laser-based SLA allow for high resolution 3D printing, but the adoption of this method in the field of MPSs is hampered by the relative high cost of the technology and the limited selection of biomaterials. However, advances made in the past few years suggest that future development of this technology could rapidly lead to a broader adoption of this method for microfabricating of anatomically relevant constructs.

The research team led by Prof. Miller has recently succeeded in establishing the first bioprinted breathing-model of the alveolar tissue, using an open-source instrument named custom-designed stereolithography apparatus for tissue engineering (SLATE) [134]. The team of Prof. Miller first optimized a photo-cross linkable hydrogel made of poly(ethylene glycol) diacrylate (PEGDA) and demonstrated the possibility of generating complex and entangled vascular networks that mimic the 3D architecture and function of human tissues. This hydrogel was shown to be compatible with several cell types, including a human fibroblast cell line (IMR90), primary human mesenchymal cells (hMSCs) and endothelial cells (HUVECs). Secondly, the team adapted a polyhedral representation of the lung alveoli to 3D print a lung biomimetic model comprehensive of distal alveolar structures reminiscent of the alveolar sacs positioned over a perfusable capillary bed (dimensions). Cyclic ventilation of the bioprinted construct with humidified oxygen gas led to significant distension of the alveolar regions. This bioprinted construct was able to withstand more than 10,000 cycles at 24 kPa and frequency of 0.5 Hz over 6 h, demonstrating physiologically relevant compliance, suggesting the potential of this construct to modelling of stress distribution during breathing motion. Finally, the perfusion of deoxygenated red blood cells (RBC) through the vascular inlet of this construct during cyclic ventilation, resulted in an increased percentage of oxygenated RBC, therefore, once again, demonstrating the relevance of mechanical cues to recapitulate tissue and organ-level functions.

The approach described here was originally developed as a functional proof-of-concept to support future applications of 3D bioprinting tissues for therapeutic transplantation. However, given the demonstrated ability to recapitulate physiologically relevant tissue-function within a microfluidic structure that closely resembles the 3D architecture of a human tissue, we believe that future advancements of this technology could be directed towards the establishment of PDMS-free, scalable, and predictive MPSs.

### 6.7. Advantages of Bioprinted MPSs

Generating MPSs via bioprinting has multiple advantages over traditional microfabrication methods, including the possibility of generating membrane-free and PDMS-free devices, precise modelling of the 3D architecture of vascularized tissues, and potential future applications in clinical settings. For example, extrusion bioprinting allows the use of heterogeneous 3D structures in a controlled extracellular environment, which can better mimic the biochemical and biomechanical properties of human TME. Bioprinted hydrogels generally consist of an ample amount of water (about 70–99%) alongside a cross-linked polymer network. The water content provides physical similarity to tissues and helps to provide excellent biocompatibility. Crosslinking agents help to stabilize the hydrogel constructs and to adjust the viscosity and stiffness of biopolymers (varying from 0.5 kPa–5 MPa), allowing the bioprinted surface to match the stiffness of native tissues, thereby capturing an important regulative element of the TME [150]. Moreover, the hydrophilic pores of biocompatible matrixes (such as hydrogels) allow small molecules to diffuse freely (like water) as opposed to PDMS where small molecules are often trapped into a hydrophobic pocket what limits the applicability of PDMS-based device in drug testing.

The possibility of creating MPSs incorporating living human cells via a “single-step” bioprinting process is another advantageous aspect that is often underestimated but relevant to reduce the in “between-lab” reproducibility occurring with traditional microfabrication methods. Bioprinting builds on technologies and engineering methods, including software and the coding language (G-code), previously developed in the field of additive manufacturing. Similar to any other 3D-printer, a bioprinter will follow the commands encoded in a G-code file to fabricate several constructs that are virtually all identical. Such automated manufacturing method facilitates a more efficient, reproducible, and automated workflow. As protocols and construct-designs become more available through free repositories of software and open-source projects, bioprinting may become a common approach for developing of new design-concepts that could further advance the field of alternative methods to animal models.

## 7. Discussion and Future Directions

While initially developed with the main goal of providing functional alternatives to animal testing, recent progress made in generating of iPSc-derived OOCs and MPSs offer new tools for personalized medicine applications. Given the rapid spread of affordable bioprinters, combined with the tremendous advances in regenerative medicine, here we speculate that in the near future patient derived OOCs and MPSs could be generated within medical facilities. These devices could function as surrogates (“Avatars”) of patient-organs designed to reflect the individual pathogenic state and response to pharmaceutical treatment.

### Bioprinted MPSs Represent a Fresh Perspective on Strategies to Develop Personalized Models of Human Diseases

Realization of a simple and yet powerful idea of personalized medicine has inspired a major progress in biomedical research over the past two decades. Significant advances in genome sequencing and in silico analysis have led to the prospect of precision medicine, where the development of new therapeutics can be advised by the genetic background of individuals. Thus, precision medicine holds the immense promise of a new and better approach to healthcare based on each person’s unique genetic profile. Despite the remarkable progress in medicine and genomics that has occurred since the completion of the human genome project, only a few personalized medicine applications have been successfully translated into clinical practice. While “omics” analysis (including genomics, metabolomics and, proteomics) has provided us with important insights into the variation of drug responses between different individuals and helped to identify genetic risk factors for a wide range of human diseases, the clinical use of omics-based predictors to guide patient therapy is extremely limited. The lack of impact on clinical practice can largely be attributed to the size and complexity of data obtained through omics and the lack of validation methods.

Due to the ability of mimicking the dynamic nature of human TMEs, stem-cell derived OOCs and MPSs holds the promise of recapitulating the pathophysiological state of tissues (or even organs) of individual patients. These devices encapsulate all the essential elements to address the current lack of validation methods in precision medicine. Unfortunately, the applicability of this technology for precision medicine is currently limited to the proof-of-principle stage and far from the reality of clinical practice.

Part of the issue is that microfabrication methods and materials used for fabricating OOCs require highly specialized personnel and expensive instrumentations. Up until a few years ago, only a handful of specialized laboratories were equipped for fabricating OOCs. Producing OOCs via traditional lithography and PDMS replication process requires dedicated clean rooms equipped with expensive instruments for fabricating silicon wafers that are used for generating PDMS stamps and microfluidic constructs. The resulting microfluidic devices need to be further treated for cell culture (surface functionalization), coated in ECM and finally seeded with human cells. Therefore, labs fabricating OOCs needs to be equipped with cell culture facilities operated by highly trained personnel, capable of both performing cell culture and handling microfluidic devices.

The widespread availability of high-resolution 3D printers has changed the ways of microfabricating microfluidic devices, allowing a dramatic reduction in costs and time needed for the building of OOCs. Recent improvements in 3D printing technology have allowed researchers to generate moulds made of affordable polymers (resins) that can be used for the direct casting of liquid PDMS. Once cured, the PDMS constructs can be transferred and used for cell culturing. It is likely that the rapid diffusion of commercial bioprinters may further simplify the process of microfabricating living MPSs. Bioprinters are instruments built to enable fabrication of biologically compatible 3D structures with a high level of automation. Cell-laden bioinks can be directly extruded into prefabricated holders and eventually perfused with cell culture medium. Different than traditional OOCs made via PDMS replication, these bioprinted devices can be built to incorporate cells living within a 3D ECM without the need of further steps such as surface functionalization and cell seeding.

Bioprinting can accelerate the “democratization” of MPSs technology via reducing the need of specialized engineering know-how, which may eventually lead to a decentralization of “Chip” manufacturing that currently occurs only in a limited number of specialized labs and companies.

Because of the vast number of medical applications, it is plausible to assume that as commercial bioprinters become more user-friendly and available to the broader biomedical community, in the coming years a growing number of clinicians will have access to these instruments. Generating these devices in a clinical setting will bring this technology closer to the patient and may facilitate the adoption and use of precision medicine in hospitals.

Another advantage of biopriting MPSs is the abundancy of cells in the clinical setting. In the past decade, the growing interest in regenerative medicine and stem cell derived therapies has resulted in the implementation of protocols for banking of iPSc-derived cells in a growing number of hospitals. Research hospitals represent a unique source of patient-derived cells for modelling genetically inherited disorders, as well as for building in vitro platforms that could help to identify (and stratify) a patient population during clinical trials.

**Figure 7:**
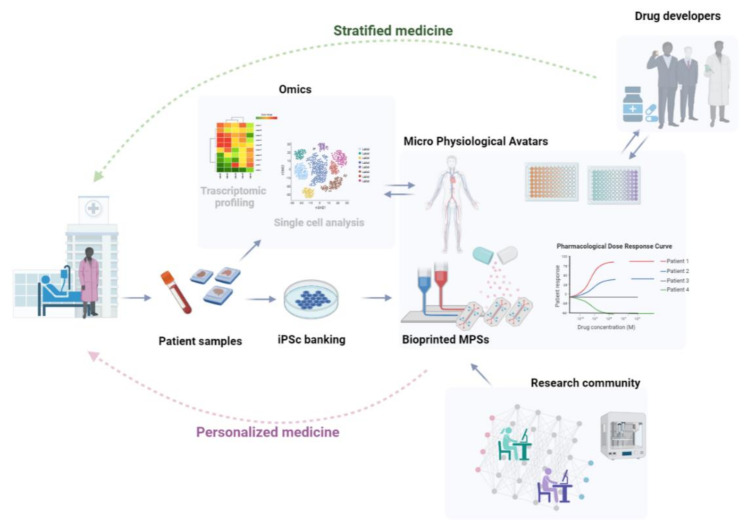
Bioprinted Microphysiological systems new tools in precision medicine. Created with BioRender.com.

We envision a future where cells obtained from diseased and healthy patients will be first (Figure 7) reprogrammed into stem cells (iPSc) and banked within the same hospital using currently existing methods and then differentiated and used for generating living MPSs designed to reflect the biology of a specific patient. These Avatars could have multiple uses, including safety assessment of drugs for precise tailoring of pharmaceutical therapies in one patient (precision medicine), identification of sub-groups of patients based on individual drug-response (stratified medicine), and conducting preclinical trial on-Chips with the goal of generating therapeutic compounds designed to treat a selected (stratified) group of patients.

## Figures and Tables

**Table 1:** Overview of advantages and disadvantages of different methods for microfabricating Organ-on-Chips.

Fabrication Method	Advantages	Disadvantages	Ref.
**Soft Lithography**	Time saving, inexpensive, reusable molds from single master, easier setup, high throughput, wide range of resolution nano to micrometer	Structure needs to be continuous, other technique such as photolithography, e beam is required to fabricate master stamp once	[76,77]
**Photolithography**	Robust, repeatable, resolution ranges from micron to millimeter	Requires expensive equipment, and specialized training, time consuming, limited to photocrosslinkable materials	[78,79]
**Etching**	High resolution, sub micrometer size, well known technique	Chemicals(acid/base) may damage the polymers, Poor process control and selectivity due to temperature gradient, multiple steps, high cost and time	[9,80]
**Laser cutting**	Robust, time saving, allows for alternative plastics that replace PDMS	Laser cutting of plastic components can be expensive as it requires the use of ventilated rooms or instruments designed to remove harmful gas.	[81]
